# Housing situations and local COVID-19 infection dynamics using small-area data

**DOI:** 10.1038/s41598-023-40734-0

**Published:** 2023-08-31

**Authors:** Diana Freise, Valentin Schiele, Hendrik Schmitz

**Affiliations:** 1 Paderborn University, Paderborn, Germany; 2 RWI Essen, Essen, Germany; 3 Leibniz Science Campus Ruhr, Essen, Germany

**Keywords:** Risk factors, Viral infection, Epidemiology

## Abstract

Low socio-economic status is associated with higher SARS-CoV-2 incidences. In this paper we study whether this is a result of differences in (1) the frequency, (2) intensity, and/or (3) duration of local SARS-CoV-2 outbreaks depending on the local housing situations. So far, there is not clear evidence which of the three factors dominates. Using small-scale data from neighborhoods in the German city Essen and a flexible estimation approach which does not require prior knowledge about specific transmission characteristics of SARS-CoV-2, behavioral responses or other potential model parameters, we find evidence for the last of the three hypotheses. Outbreaks do not happen more often in less well-off areas or are more severe (in terms of the number of cases), but they last longer. This indicates that the socio-economic gradient in infection levels is at least in parts a result of a more sustained spread of infections in neighborhoods with worse housing conditions after local outbreaks and suggests that in case of an epidemic allocating scarce resources in containment measures to areas with poor housing conditions might have the greatest benefit.

## Introduction

By the end of 2022, the World Health Organization (WHO) registered more than 656 million SARS-CoV-2 (severe acute respiratory syndrome Corona virus type 2) cases worldwide^1^, with the number of confirmed cases representing only a fraction of the actual cases^2^. Since the beginning of the COVID-19 pandemic, researchers of various disciplines investigate the spread of the virus as understanding this may help to develop strategies to cope with future epidemics. In this paper, we use small-scale data (see below for an explanation of the term small-scale) to study outbreak patterns of SARS-CoV-2. In particular, we focus on the role of the residential environment in order to learn what role it plays in the frequency, intensity and duration of local outbreaks and, thus, in the spread of the epidemic. This may help in the future to allocate scarce resources in containment measures to the areas where they will have the greatest benefit.

Several literature reviews address previous research on the spread of SARS-CoV-2 and contextualize the factors analyzed, the region and timing, and the methodology used. Most of the literature to date uses spatial and spatio-temporal methods in most cross-sectional and ecological studies, not only to investigate clusters in the spread or to simulate its evolution, but also to understand the role of socio-economic status and associated determinants in driving dispersal (see, for example, Franch-Pardo et al.^3, 4^ and Nazia et al.^5^ for more information on the methodology used in this context). Alidadi and Sharifi^6^ review the literature examining the human (made) factors (for instance, density, transportation and mobility, demographic and socio-economic factors) responsible for the spread. They identify a total of seven important fields, one of which is housing conditions. Khanijahani^7^ focuses on literature examining in particular racial and ethnic as well as socioeconomic disparities in the pandemic and, thus, reviews parts of this literature strand. Since a full review of this literature is beyond the scope of this article, we summarize the main findings of these studies below, and refer the interested reader to the above-mentioned literature reviews for a more exhaustive overview.

Studies show differences in the first phase of the pandemic compared to following waves. In Germany, for instance, the first infections spread particularly in more affluent areas, where infections occurred through travelers from skiing holidays and other international travels. Socially more deprived districts were less affected at this time. This changed in the second phase, where districts with higher educated people had lower incidence rates^8^. This is confirmed by Rohleder et al.^9^ who also find higher deprivation as well as non-nationality to be positively correlated to a higher incidence risk, except for the first wave. Similar relations are also observed in the United states^10^, and cities like Barcelona^11^, Helsinki^12^ or Rio de Janeiro^13^. In addition, Hoebel et al.^14^ observe higher mortality for socio-economically deprived German districts. These findings already suggest housing conditions to play a role in the infection process, as deprivation, housing and health are strongly linked to each other and are not trivial to be disentangled^15^. While both urban density and connectivity within or between cities contribute to the spread, the latter one is better indicator^16^. Therefore, with the need for social distancing especially in the first phase of the pandemic, this is probably easier for people in better housing situations allowing also for home office, isolation of infected individuals and more testing opportunities. The role of housing characteristics in its variations is therefore of particular interest in the understanding of COVID-19. Ahmad et al.^17^ find higher incidence and mortality rates for US counties characterized by a higher percentage of households with severe housing problems, including overcrowding, high housing cost burden, incomplete kitchen facilities, and incomplete plumbing facilities. Besides overcrowding (see also Lee et al.^18^), areas with a higher proportion of multigenerational households are associated with higher incidence rates^19^. Overcrowded homes are not only associated with higher incidence, but also mortality^20^.

We contribute to this literature in the following way. While the focus of many of these studies lies on the differences in levels of infection rates and deaths by socioeconomic status, we study *the evolution of local SARS-CoV-2 outbreaks* on a rather disaggregate level. This might help to understand how the commonly observed differences in infection *levels* across neighborhoods are shaped. Specifically, we ask whether local outbreaks are *(a)* more frequent, *(b)* more severe, and *(c)* longer lasting in disadvantaged areas compared to more affluent areas. To study patterns of local SARS-CoV-2 outbreaks, we combine small-scale district-level infection data with small-scale housing quality data for the city of Essen, a major city in the west of Germany with nearly 600.000 inhabitants and 50 local districts (“Stadtteile”), and rely on event-study graphs allowing to visualize outbreak trajectories and potential differences by residential environment. Event study methods are increasingly used in the literature on COVID-19/SARS-CoV-2, e.g. to study the impact of policy interventions on the spread of the epidemic (see e.g. Askitas et al.^21^, Bárcena-Martín et al.^22^, Bertocchi and Dimico^23^ and Dave et al.^24^), due to their flexibility, simplicity and transparency. Their major advantage in studying outbreak trajectories, also compared to epidemiological models, is that their use does not require prior assumptions about transmission characteristics of SARS-CoV-2, the immune status and susceptibility, or behavioral responses of the population (all of which changed during the pandemic). Our results show that local outbreaks are not more frequent or severe in less affluent districts. However, while they tend to end more than 20 days after the onset of the outbreak in districts with better housing, cases are higher in less affluent districts even after at least 25 days, suggesting sustained transmission of infections in these districts also in the longer run. This finding may not only explain the socioeconomic disparity in infection rates found in previous studies, but also calls for a greater focus of interventions such as local vaccination initiatives or information campaigns in neighborhoods with poor housing conditions.

## Data

For our analysis we combine small-scale data from different data sources. The unit of observation is the local district (“Stadtteil”) in Essen, a large city in Western Germany with nearly 600.000 inhabitants and 50 local districts. Essen has an area of 210 square kilometers, thus, the average size of the 50 local districts is around four square kilometers (or two by two kilometers). This is typically called “small-scale” or “small-area” as it is much smaller than, for instance, the county level (“Kreis”) for which German infection data are typically available with the largest county having a size of 74 by 74 kilometers. SARS-CoV-2 incidence rates—which are the numbers of new cases recorded per 100,000 people in seven days—are provided to us by the city of Essen. These are also used to calculate the number of cumulative cases (per 1000 inhabitants). We use daily data from 1 March 2020 to 31 December 2021, that is, 50 times 671 data points on infection rates. Information on housing situation is provided by the FDZ Ruhr. The data is based on different sources: the Federal Agency for Cartography and Geodesy (information on the quality of the residential area), ImmoScout24—the leading online platform for residential and commercial real estate in Germany—(information on the rent index) and the Federal Statistical Office of Germany (information on household sizes), see^25^.

**Figure 1:**
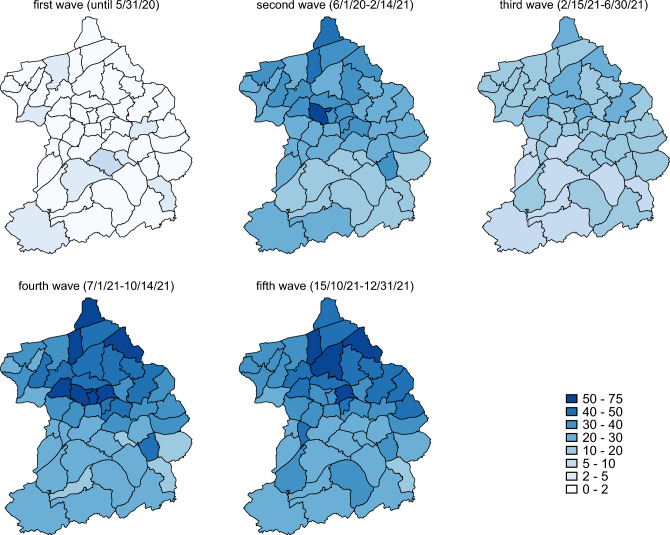
Illustration of the regional variation of cumulative cases between local districts. Each map shows cumulative cases per 1000 inhabitants for a specific time frame. The figure was created using Stata 17 (https://www.stata.com/).

Figure 1 illustrates the regional variation of cumulative cases per 1000 inhabitants within the city and over the course of the pandemic until the end of 2021. With the mutation to the Omicron variant and less severe progression of Covid infection, official data that only count positively tested individuals became less reliable when less and less people with infections decided to get tested. Therefore, we only analyze outbreaks up to this point in time. The classification of the waves is based on the incidence development in Essen. Figure S1 in the Supplementary Information shows the incidence rate in Essen until 31 December 2021. According to our classification, we declare a wave to be over as soon as it reaches a low point after flattening out. In Fig. 1, it is noticeable that the north of Essen has more cases, with the exception of the first wave where the first cases occurred predominantly in the wealthier districts.

**Figure 2:**
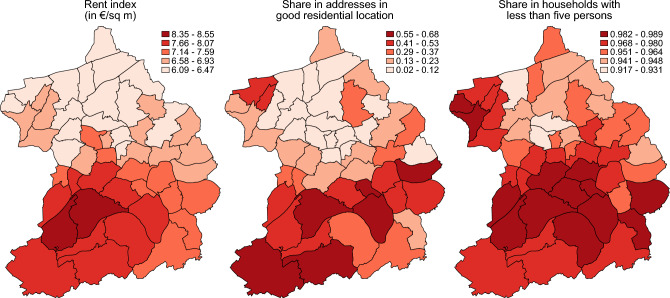
Illustration of the regional variation in housing characteristics between local districts. Classification is based on k-means partition cluster analysis. The figure was created using Stata 17 (https://www.stata.com/).

The regional differences in Essen—a city with a strong North–South-divide—also become apparent in the consideration of residential factors in Fig. 2. The left map shows the regional variation in the rent index (in euros per square meter) which reflects the local rent. More expensive neighborhoods are prevalent in the south.

To map the location quality of the residential environment, we look at the share of addresses that are classified as good residential locations. Good residential locations are characterized by open and green spaces, a well-kept street scene and very good transport connections, good shopping possibilities and a good image. The middle map shows the regional variation in the share of addresses in good residential location, ranging from less than 13 percent in northern local districts to more than 55 percent in some southern local districts. We provide additional information on how this measure is constructed in Sect. S2 of the Supplementary Information.

Less crowded households may be less prone to Corona infections. We calculate the share of households with less than five persons—in this sense less crowded households—and see in the right map of Fig. 2, that more than 96 percent of households consist of less than five persons in most districts in the south of Essen. This share is slightly lower in the northern local districts.

For the main analysis, we concentrate on these characteristics and divide each into terciles. Thus, the first tercile of the rent index contains a third of the districts with the lowest rent level. The first tercile of good residential location contains a third of the districts with the lowest share of addresses in good location and the first tercile considering the household size contains a third of the districts with the lowest share in households with less than five persons. Table 1 summarizes descriptive statistics for these housing characteristics where especially the mean differences in terciles for good residential location are apparent. The mean rent index ranges from around 6–8.5 euros per square meter. The average rent in a district from the group of 33.33% of districts with the lowest rent level, is 6.3 euros per square meter whereas in the group of 33.33% of districts with the highest rent level, it is 7.82 euros per square meter on average. The share in addresses in good location ranges from around 1.5 percent to over 67 percent. The difference in mean shares of the first and third tercile are in this respect more obvious. In districts with the lowest proportion of good location, on average every fifth address is in good quality, whereas in districts with the highest proportion of good location it is one in two addresses. The differences in shares in households with less than five persons ranges from around 92–99 percent.

**Table 1:** Descriptive statistics for housing characteristics.

	Mean	SD	Min	1st terc.	2nd terc.	3rd terc.	Max
Rent index (in €/sq m)	7.01	0.67	6.09	6.30	6.91	7.82	8.55
Addresses in good location (in %)	26.64	20.04	1.53	5.68	24.48	51.19	67.68
Households < 5 (in %)	96.62	1.82	91.69	94.46	97.06	98.46	98.86

When comparing Figs. 1 and 2, infections and housing situations seem to be linked. To quantify the graphical impression that there is a relationship between Corona infections and housing situation and to estimate the relative significance of the individual indicators, we run bivariate OLS regressions of cumulative cases per 1000 inhabitants on each characteristic, separately for each identified Corona wave in Essen. These regressions are intended to replicate the correlations from the existing literature. Although the data does not contain the necessary information to obtain causal estimates by including additional variables, the selected measures rather serve as indicators to reflect more general differences in housing situations. Table 2 shows standardized coefficient estimates. Except for the first wave (column (1)), the relationship between cumulative cases and housing characteristics is negative which is also in line with the previous discussed literature. One standard deviation increase in the rent index (and respectively addresses in good location or households with less than 5 persons) goes along with a 0.305 (and respectively 0.245 or 0.312) standard deviations increase in cumulative cases at the end of wave 1. With reference to columns (2)–(5), the relationships are throughout negative and range between 0.4 and 0.8 standard deviations decrease in cumulative cases. All in all, except for wave 1, local districts with higher rents, higher shares of addresses in good location and less crowded households seem to be less prone to Corona infections. The relationship is very similar in waves 2–4 but somewhat smaller in wave 5. A speculative reason for the less strong relationship in wave 5 is that at that time immunization rates of individuals in worse housing conditions had become better due to higher infection rates before. Yet, it is beyond the scope of the paper to scrutinize this claim.

**Table 2:** Bivariate regression results of cumulative cases on housing characteristics.

Dep. var.: Cumulative cases (per 1000 inhabitants) from current wave					
	(1)	(2)	(3)	(4)	(5)
	Wave 1	Wave 2	Wave 3	Wave 4	Wave 5
Rent index (in euros/sq m)	0.305$$^{**}$$	$$-0.562^{***}$$	$$-0.789^{***}$$	$$-0.684^{***}$$	$$-0.416^{***}$$
	(0.137)	(0.119)	(0.089)	(0.105)	(0.131)
Addresses in good location (in %)	0.245$$^{*}$$	$$-0.567^{***}$$	$$-0.518^{***}$$	-0.638$$^{***}$$	$$-0.400^{***}$$
	(0.140)	(0.119)	(0.123)	(0.111)	(0.132)
Households < 5 (in %)	0.312$$^{**}$$	$$-0.761^{***}$$	$$-0.697^{***}$$	$$-0.735^{***}$$	$$-0.470^{***}$$
	(0.137)	(0.094)	(0.104)	(0.098)	(0.127)
$N$	50	50	50	50	50

Coefficients estimates are standardized. Standard errors in parentheses. *$p < 0.1$, **$p < 0.05$, ***$p < 0.01$. Figure S6 in the Supplementary Information shows the joint distribution of cumulative cases and housing conditions by wave and for each of the three housing measures.

## Methods and results

### Methods

The analysis so far shows a clear association between housing situation and SARS-CoV-2 incidence. In all waves except for the first, districts with a higher proportion of good housing conditions have significantly fewer cases than neighborhoods characterized by low rents, a low proportion of households in good residential location, and large household sizes. This suggests that the frequency, intensity, and duration of local outbreaks might depend on the residential environment. A potential explanation for this finding is that more affluent individuals may find it easier to reduce their personal risk of infection following a Corona outbreak in their community through social distancing and hygiene measures (home office, isolation of infected individuals in the household, regular testing, wearing KN95/FFP2 masks, etc.) than individuals living in more precarious residential areas. This, in turn, could contribute to a rapid stagnation or even decline of incidence in one neighborhood after an outbreak, whereas it may rise for a longer period of time in another and only decline later. We now change the time dimension in our analysis from *wave* to *day* and exploit the daily information on 7-day Corona incidence. Using event studies, we will test the hypothesis that the trajectories of local outbreaks differ by the residential environment. This requires to define what constitutes a local outbreak and its onset in a first step.

#### Identification of local Corona outbreaks

One way to define the onset of a Corona outbreak, which seems natural at first glance, would be to use the occurrence of a first case after a predefined period of time without active cases in the respective district. However, in view of the wide spread of SARS-CoV-2 from autumn 2020 at the latest and the associated permanent transmission of individual infections from one area to the other, such an approach is not very purposeful. Therefore, and since there is no generally accepted definition of local Corona outbreaks, we use a data-driven approach in this paper to identify (the onset of) local outbreaks. Broadly speaking, an outbreak is defined here as a sharp increase in the Corona incidence over a period of at least 7 days after a stagnation of the incidence over a period of at least 7 days. In our preferred specification, stagnation corresponds to an average change in daily Corona incidence in the range of +-2 cases per 100,000 inhabitants. A sharp increase in incidence then corresponds to an increase in active cases by an average of at least 15 cases per 100,000 inhabitants per day and this for a period of at least 7 days. Technically, we identify (the onset of) outbreaks by running a series of linear regressions. For each district *i* and day *k* we fit the following two linear regressions to the data

1 $$\begin{aligned} y_{it}&=\alpha _{1ik} + \beta _{1ik} t + \varepsilon _{1it} & \text {for } k-7<t<k \end{aligned}$$

2 $$\begin{aligned} y_{it}&=\alpha _{2ik} + \beta _{2ik} t + \varepsilon _{2it} & \text {for } k< t<k+7 \end{aligned}$$

where $y_{it}$ denotes the 7-day Corona incidence (the sum of new Corona infections in the previous seven days) in district *i* on day *t*; $\alpha _{1ik}$ and $\alpha _{2ik}$ are intercepts and $\beta _{1ik}$ and $\beta _{2ik}$ are the slope parameters of the of the rolling window regressions. This yields $k = 1, \dots , 657 (=671-7-7)$ estimates for $\beta _{1ik}$ and $\beta _{2ik}$ for each of the $i=1,\dots , 50$ districts. Whether day $t=k$ marks the starting point of a Corona outbreak in district *i* then follows from a comparison of the slope parameters belonging to this day with the corresponding threshold values. Day *k* only marks the onset of an outbreak if $-2<\beta _{1ik}<2$ and $15<\beta _{2ik}$, i.e. if the incidences in the 7 days before day *k* have not changed by more than 2 cases per day and 100.000 inhabitants on average and the incidences in the 7 days following day *k* have increased on average by at least 15 cases per day and 100.000 inhabitants.

**Figure 3:**
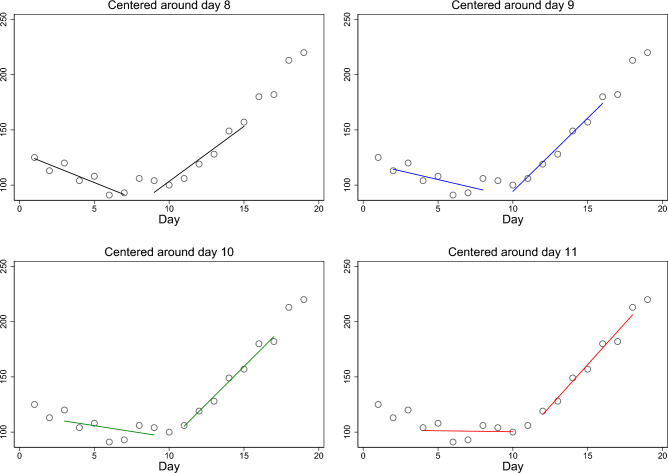
Stylized example of local Corona outbreak identification for one district. The vertical axis measures the 7-day incidence. The figure was created using Stata 17 (https://www.stata.com/).

Figure 3 provides a stylized example that illustrates our approach to identify local Corona outbreaks. It shows simulated daily incidences for the arbitrarily chosen days $t=1,\ldots ,19$ in a hypothetical district, along with the fitted regression lines for varying values of *k*. In the upper left panel, the center of the rolling estimation window is day 8, i.e. $k=8$ and accordingly the first regression line is fitted through the data for days 1–7 and the second through the data for days 9–15. In the next panel, the center of the estimation window is moved one day ahead, and the regression lines are fitted through the data for the days 2–8 and 10–16, respectively, and so forth. By comparing the slope parameter of the regression lines to the respective thresholds (i.e. $-2<{\widehat{\beta }}_{1ik}<2$ and ${\widehat{\beta }}_{2ik}>15$), we are then able to identify the onset of an outbreak. In Fig. 3, we observe a slope parameter in all regression lines left of the center of the rolling window smaller than $-2$ and a slope parameter smaller than 15 in all regression lines right of the center for all panels except for the one in the lower right. Only on day 11, we observe a clear stagnation of cases in the 7 days before and a clear increase (>15 cases per day and 100,000 inhabitants) during the following 7 days. Thus, based on our approach for identification of local outbreaks, we would conclude that day 11 marks the start of the local outbreak in our hypothetical district.

In Fig. S2 in the Supplementary Information, we show a selection of outbreaks using the actual data from Essen, both from more and less affluent districts and at different points in time, along with the estimated regression lines that identify the start date of the respective outbreak. Table S1 in the Supplementary Information gives an overview of the number of local outbreaks identified in the data using this approach. In total, we identify 167 local outbreaks in the 50 districts of the city of Essen during the observation period. Most of these outbreaks occur in the second and fiftth wave, several in waves 3 and 4, and only a negligible number in the first wave. Given that the thresholds for our outbreak definition are chosen somewhat arbitrarily, we also provide results for two alternative definitions where we use other thresholds and show that the results of our analysis are not sensitive to the exact definition of outbreaks. Table S1 also reports the thresholds and number of outbreaks identified using these alternative outbreak definitions.

#### Event study estimations

Based on the definition of Corona outbreaks and all local outbreaks identified in the data, we can analyze and visualize the courses of the outbreaks in order to investigate whether they differ depending on the residential environment. For this purpose, we use event-study graphs in the following, which plot the development of incidences as a function of the temporal distance to the onset of the local Corona outbreak.

We define the event time *r* as the time (in days) between a day and the day at which a local outbreak starts in district *i*:

$$\begin{aligned}r_{it} = t - e_{i}\end{aligned}$$

where *t* denotes calender time in days, *i* is the district, and $e_{i}$ denotes the day that has been identified as the day when a local outbreak starts in district *i*. Therefore, $r = -1$ is the last day before the onset of the outbreak, $r = 0$ is the day of the onset of the outbreak and $r = 1$ is the first day after the onset of the outbreak and so on. Based on this definition of relative time, we run the following baseline fixed effects regression:

3 $$\begin{aligned} y_{it} = \sum \limits _{\begin{array}{c} j=-10 \\ j\ne -1 \end{array}}^{24} \mu _j 1[r = j] + \alpha _i + \lambda _m + \tau _w + \varepsilon _{it} \end{aligned}$$

where $y_{it}$ denotes the 7-day incidence on day *t* in district *i*, that is, the sum of new Corona infections in the previous seven days. The indicator function $1[r = j]$ takes on the value 1 if $r = j$ and 0 otherwise. That is, we include a full set of binary variables that non-parametrically account for the relative time *r* (i.e. of the event time). $\mu _j$ (where $j = -10, \ldots 24$) are the 35 coefficients of the indicator variables. We restrict $\mu _{-1}=0$ and trim the sample to the left at $j<-10$. For $j>0$ we include all event-time indicators and report them up to day 24 after the start of an outbreak. $\alpha _i$, $\lambda _m$, and $\tau _w$ are district, calender month and wave fixed effects. The 7-day Corona incidence is—other than the reported new cases in Germany - not sensitive to the day of the week. Therefore, day of the week fixed effects are not included. By plotting the resulting event time coefficients against the relative distance to the start of an outbreak, we can empirically evaluate how cases evolve over the course of a local outbreak without making prior assumptions about the transmission properties of SARS-CoV-2 or the susceptibility of the population. Splitting the sample by residential characteristics then allows to study potential differences in outbreak patterns by housing conditions.

### Results

**Figure 4:**
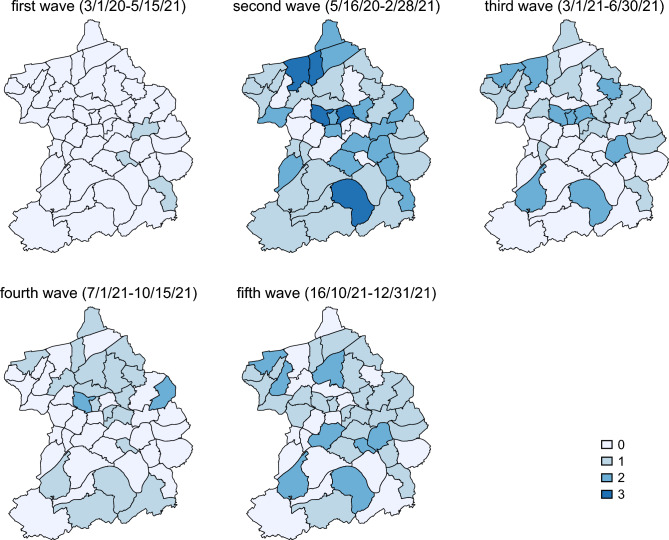
Number of outbreaks in neighborhoods. The figure was created using Stata 17 (https://www.stata.com/).

Before turning to the event study results, Fig. 4 shows the geographical distribution of outbreaks by wave to assess whether districts with bad housing conditions experience Corona outbreaks more often. Local outbreaks can be observed in almost all districts at some point in time. In wave 3 and 4, it seems that local outbreaks happen more frequent in bad residential locations in the north while this is not the case in waves 1, 2 and 5. Overall, incidence of Corona outbreaks appear to be across all areas.

**Figure 5:**
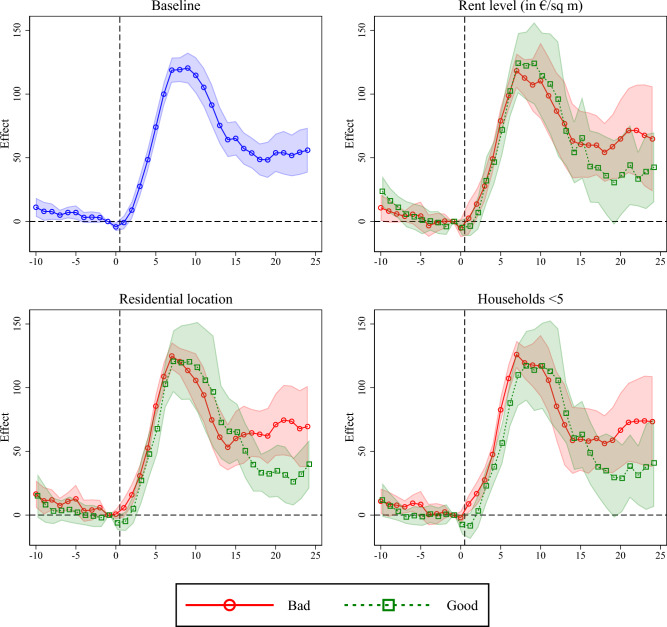
Event study results. Coefficients corresponding to $\mu _j$ in Eq. 3. $\mu _{-1}$ is restricted to zero. 95% confidence intervals reported. Standard errors clustered on district level. The figure was created using Stata 17 (https://www.stata.com/).

To shed light on the trajectories of local outbreaks and address the question whether local Corona outbreaks are also more intense and longer in districts with bad housing conditions, we now turn to the event study results. Figure 5 plots the point estimates for $\mu _j$, derived from estimating Eq. 3, along with 95% confidence intervals against relative time *r* for four specifications. The upper left panel shows baseline results for the entire sample, the following panels provide separate results by housing condition. Here, we distinguish between districts with high (in the top tercile) versus low (in the bottom tercile) shares of good housing, using rent levels as an indicator in the top right panel, housing locations in the bottom left panel, and household size in the bottom right panel (see “Data” section for details on definition of variables). That is, here we perform separate subsample analyses by districts with bad/good locations as defined by the three different measures of housing condition we use. The baseline results show that after a stagnation or slight decline in Corona incidence, cases initially rise sharply with the onset of a local outbreak. While this is not surprising given the way we define and identify outbreaks in the data, the results are still interesting. They show that the increase in case numbers accelerates during the first three to four days, but then quickly levels off. By the eighth or ninth day, the local outbreak has already peaked. Incidences then average at about 120 cases per 100,000 inhabitant above the level before the outbreak began. In the following approximately ten days, incidences fall again by about half but then tend to stagnate until at least day 24 after the start of the outbreak. On the one hand, this indicates that local outbreaks end relatively quickly, but on the other hand, it also shows that these outbreaks seem to lead to persistently higher levels of transmission of infection and thus sustain the spread of SARS-CoV-2. Interestingly, this seems to be especially true for less affluent residential areas.

The remaining panels of Fig. 5 show, that the patterns of local Corona outbreaks in less affluent residential areas are initially almost identical to those in better areas. In both less affluent and better residential areas, local outbreaks reach their peak at the same time. Up to this point, there is also no difference in the level of incidence growth. After the case numbers have reached their peak, they initially decline uniformly in both the less affluent and the better residential areas, but then diverge from around day 15 after the start of the outbreak. While the incidences in better districts seem to continue to decline on the following days, they stagnate from day 15 onwards on a higher level in less affluent districts. Although this difference is not particularly large and not statistically significant, it shows up consistently in all analyses.

**Figure 6:**
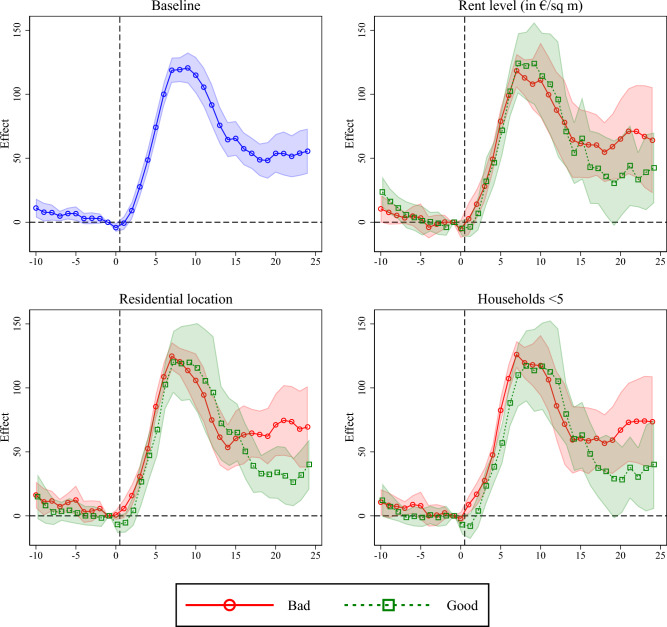
Event study results. Coefficients corresponding to $\mu _j$ in Eq. 3. $\mu _{-1}$ is restricted to zero. Regressions additionally account for (cumulative) cases up to day $t-15$. 95% confidence intervals reported. Standard errors clustered on district level. The figure was created using Stata 17 (https://www.stata.com/).

While our approach of using event studies to identify and depict outbreak patterns requires few assumptions, it can be criticized for leaving many degrees of freedom as to the definition of a local outbreak. We thus show in Figs. S3 and S4 in the Supplementary Information that the results are largely robust to the use of alternative definitions of outbreaks that are described in Table S1.

Another potential concern with our approach is that it does not take into account the level of infections of SARS-CoV-2 from which the outbreaks start and ignores earlier outbreaks. Both the infection level at the start of a local outbreak and whether there have been previous outbreaks in the same district are likely to have an impact on the intensity and duration of outbreaks, as both influence the number of potentially susceptible individuals. If this number also differs between bad and good neighborhoods, it would be misleading to attribute differences in outbreak trajectories directly to differences in the residential environment. To deal with this concern, Fig. 6 repeats the analysis but now includes the cumulative number of cases since the beginning of the pandemic (time lag of 15 days) as an additional control. In fact, the differences in outbreak trajectories between districts with good and districts with poor housing environments reduce somewhat when controlling for previous infection activity, as Fig. 6 shows. Nevertheless, there are still differences towards the end of the outbreak, suggesting that local outbreaks in poor neighborhoods lead to somewhat higher incidences in the longer term than those in good neighborhoods.

Figure S5 shows event study results for alternative measures of crowding and affluence that support or interpretations of the main results. We provide descriptive statistics and explanations for these measures in Table S2.

## Summary and conclusion

In this paper, we examine local outbreak patterns of SARS-CoV-2, focusing on the role of housing conditions for the frequency, intensity, and duration of local outbreaks. Using small-scale infection and housing quality data, we show that, in the city of Essen, Germany, local outbreaks did not occur significantly more frequently in areas with rather poor housing conditions. We also find almost no differences in the intensity of outbreaks (measured as the speed and magnitude of outbreaks) between poorer and better neighborhoods. However, our results suggest that outbreaks in more affluent neighborhoods decline further after reaching their peak than in less affluent neighborhoods, where they last longer on a higher level. This suggests that outbreaks lead to higher post-outbreak incidences and thus higher transmission rates in less affluent neighborhoods, which might explain, at least to some extent, the socioeconomic gradient in prevalence and mortality of SARS-CoV-2. While it has been found before that worse housing situations are correlated with higher incidences (see the cited studies in the introduction), we believe that it is (to the date of writing this paper) a new result in the literature that this correlation seems to be due to longer durations of local outbreaks and not due to higher frequencies of outbreaks or higher intensities.

We are aware that this is the result of one case study in a German city only, but, taken at face value it has some implications. A common hypothesis for the well-known socio-economic gradient in SARS-CoV-2 infections is that people in disadvantaged areas have less possibilities for home-office, thus higher infection rates at the workplace. Yet, given that many individuals work in other districts and that frequencies of infection do not significantly vary by districts, this does not seem to be the most important reason. Instead, once the virus enters the district, it stays longer in disadvantaged areas, implying that transmissions are more likely in the private environment, partly induced by bad housing situations like overcrowded places. Yet, obviously this a just an interpretation that is consistent with the statistics and more evidence is needed to completely back that.

For policymakers it seems relevant to visit and address individuals in disadvantaged areas much more directly regarding, for instance, local vaccination initiatives and education of effectiveness of masks. Not because individuals in these districts are less willing to vaccinate or wear masks but because they have less other options to protect themselves.

### Supplementary Information

Supplementary Information.

## Data Availability

Restrictions apply to the availability of the data that support the findings of this study, which were used under license for the current study, and so are not publicly available. The data are however available upon reasonable request for scientific purposes from the corresponding author Hendrik Schmitz (hendrik.schmitz@uni-paderborn.de).
