# Supplementary material for: Housing situations and local COVID-19 infection dynamics using small-area data
Source: Sci Rep. 2023 Aug 31;13:14301. doi: 10.1038/s41598-023-40734-0 (PMC10471764; doi:10.1038/s41598-023-40734-0)
Supplement: Supplementary file 1 — Supplementary Information. [file 41598_2023_40734_MOESM1_ESM.pdf]

S1 Additional Tables and Figures

Table S1

	Main definition: $\|\widehat{\beta}_{1ik}\| <2$ $\widehat{\beta}_{2ik} >15$	Alternative 1: $\|\widehat{\beta}_{1ik}\| <3$ $\widehat{\beta}_{2ik} >20$	Alternative 2: $\|\widehat{\beta}_{1ik}\| <5$ $\widehat{\beta}_{2ik} >30$
Wave	Number of outbreaks		
1	3	2	2
2	65	40	18
3	36	24	13
4	22	12	5
5	41	37	23
Total	167	115	61

Identified outbreaks by wave. The first column shows the number of local outbreaks by wave for the main definition of local outbreaks as outlined in Equations (1) and (2) (average daily increase in incidence of at least 15 cases per 100,000 for at least 7 days, after incidence has not changed by less than 2 cases per 100,000 and day over a period of 7 days). The following columns show the number of outbreaks for two alternative definitions that require a larger increase in cases at the beginning of an outbreak but use a less restrictive definition for case stagnation before the outbreak.

Figure S1

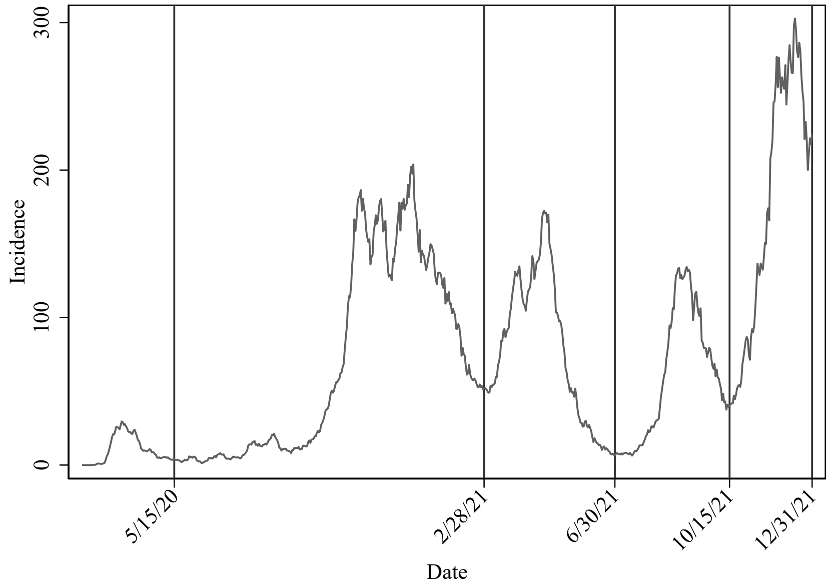

Devision of the pandemic in Essen into waves. The vertical lines mark the finish dates of waves in Essen. The figure was created using Stata 17 (https://www.stata.com/).

Figure S2

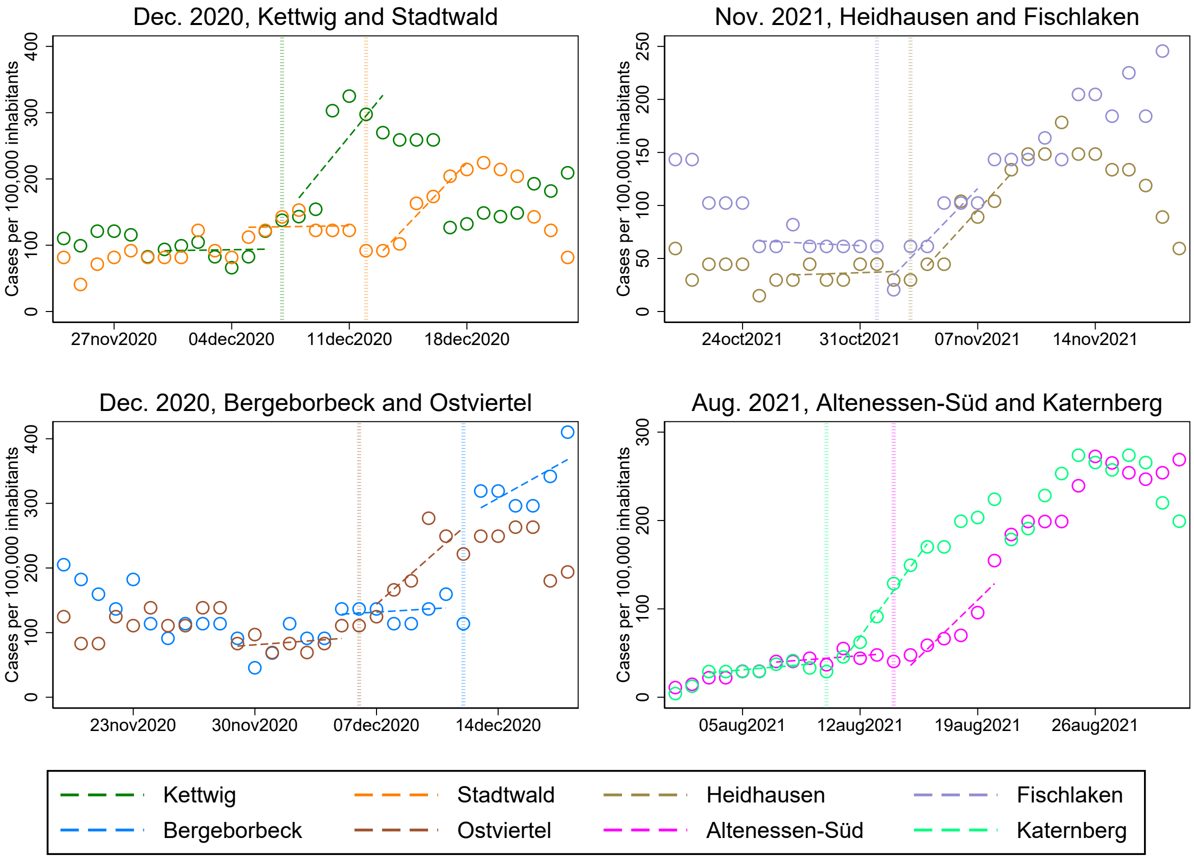

Outbreak definition for selected outbreaks. The vertical lines mark the identified start date of the respective outbreak. The dashed lines show estimated regression lines based on Equation (1) and (2). The upper two panels of the figure show selected outbreaks in more affluent districts, the lower two panels in less affluent district. The figure was created using Stata 17 (`https://www.stata.com/`).

S2 Additional information on measuring good residential location

The data provided by *infas 360* includes nine categories, each of which indicates the number of addresses with a different residential area quality. Of these, we combine three variables each to distinguish between three categories: simple (rather simple, simple and very simple), medium (medium to rather good, medium, medium to rather simple) and good (very good, good, rather good) residential areas. For each district, we then calculate the share in addresses in good residential location relative to the total number of addresses for each district.

S3 Results for alternative outbreak definitions and measures

This section provides results for alternative outbreak definitions and additional neighborhood characterists. Table S2 summarizes descriptive statistics for additional neighborhood characteristics. Figures S3 and S4 show event study results for the main measures of housing situations together with the four additional neighborhood characteristics for the alternative outbreak definitions described in Table S1. Together with the event study results for the main outbreak definition in Figure S5, the additional neighborhood characteristics (crowding, unemployment rate, migration share and purchasing power) confirm the results across the board quite well.

Table S2

	Mean	SD	Min	1st terc.	2nd terc.	3rd terc.	Max
Crowding (in residents/100 sq m)	1.16	1.92	0.24	2.20	0.81	0.52	14.18
Purchasing power (in €/resident)	22019.84	4059.40	17122	18715.65	20621.82	27015.94	34961
Unemployment rate (in %)	11.03	3.36	6.27	14.91	10.46	7.52	19.72
Migration share (in %)	29.01	12.04	11.16	43.03	27.21	17.02	60.06

Descriptive statistics for additional neighborhood characteristics. Crowding (measured in residents per 100 square meter) is calculated by dividing the number of residents in a district by the local area (measured in 100 square meter) used as residential area only. The ratio approximates how densely residents live in one spot." Purchasing power is measured in euros per resident. The district level unemployment rate and migration share are measured in percent. Similar to the variables in the main analysis, we divide each of the four alternative measures into terciles, while the first tercile refers to those districts that are assumed to be more prone to infections. Information is provided by *infas 360*. The data from *infas 360 GmbH* is based on different sources: the Federal Statistical Office of Germany (information on number of residents and migration share), the Federal Agency for Cartography and Geodesy (information on area of the districts), Basis-DLM (information on proportion of residential land), ImmoScout24 – the leading online platform for residential and commercial real estate in Germany – (information on purchasing power) and the Federal Employment Agency of Germany (information on unemployment rate).

Figure S3

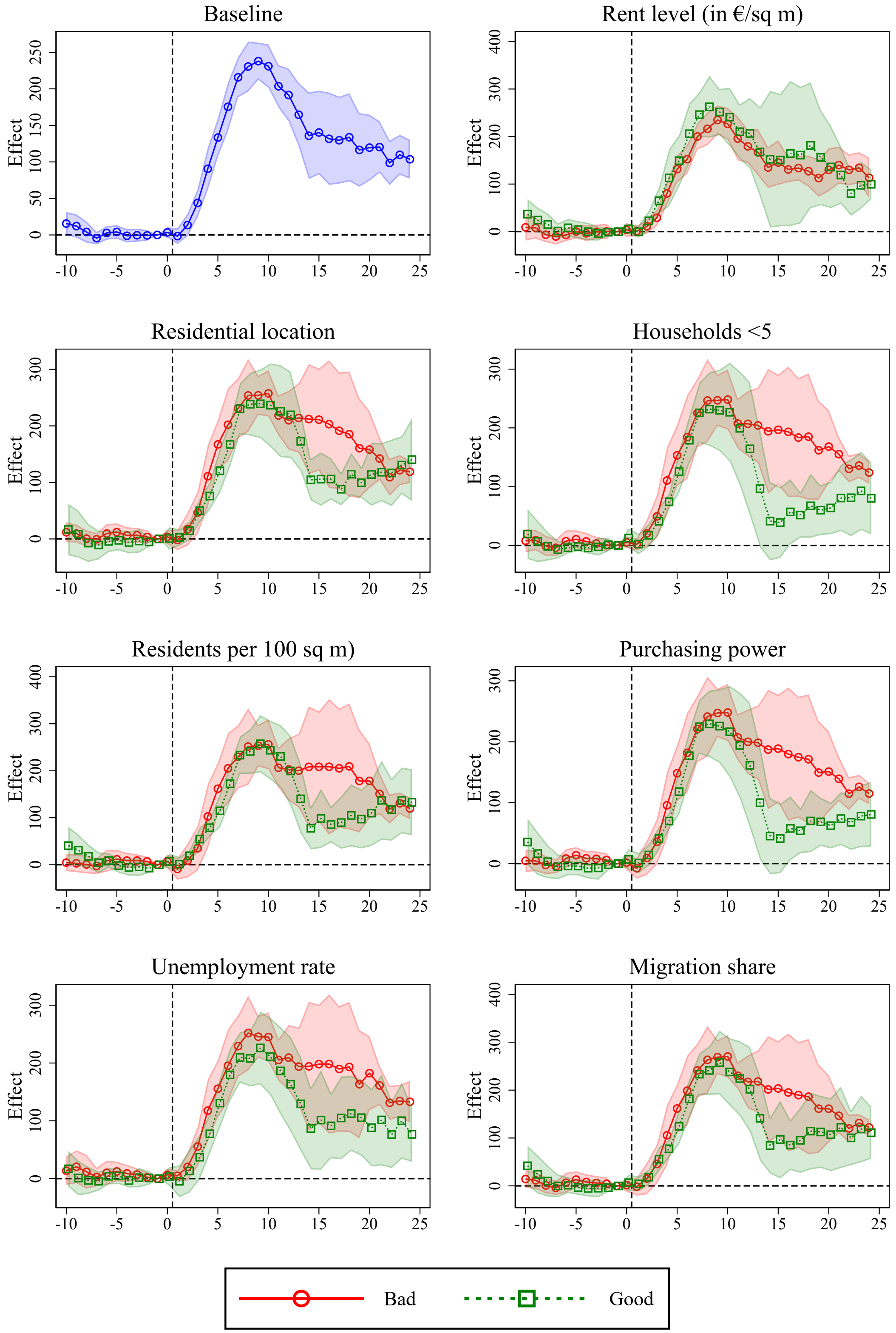

Event study results – Alternative outbreak definition 1. Coefficients corresponding to μ_j in Equation 3. μ_{-1} is restricted to zero. 95% confidence intervals reported. Standard errors clustered on district level. The figure was created using Stata 17 (https://www.stata.com/).

Figure S4

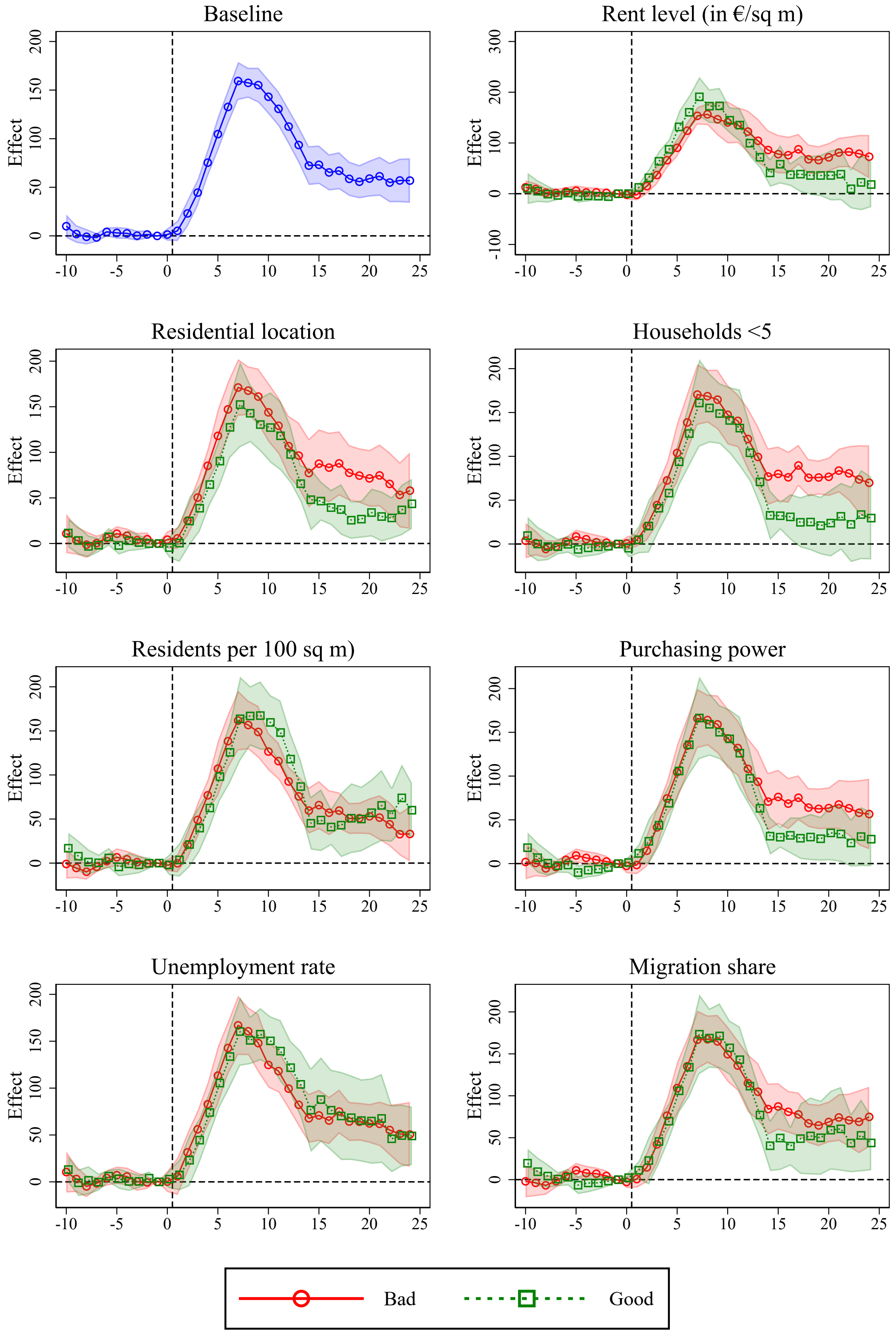

Event study results – Alternative outbreak definition 2. Coefficients corresponding to μ_j in Equation 3. μ_{-1} is restricted to zero. 95% confidence intervals reported. Standard errors clustered on district level. The figure was created using Stata 17 (https://www.stata.com/).

Figure S5

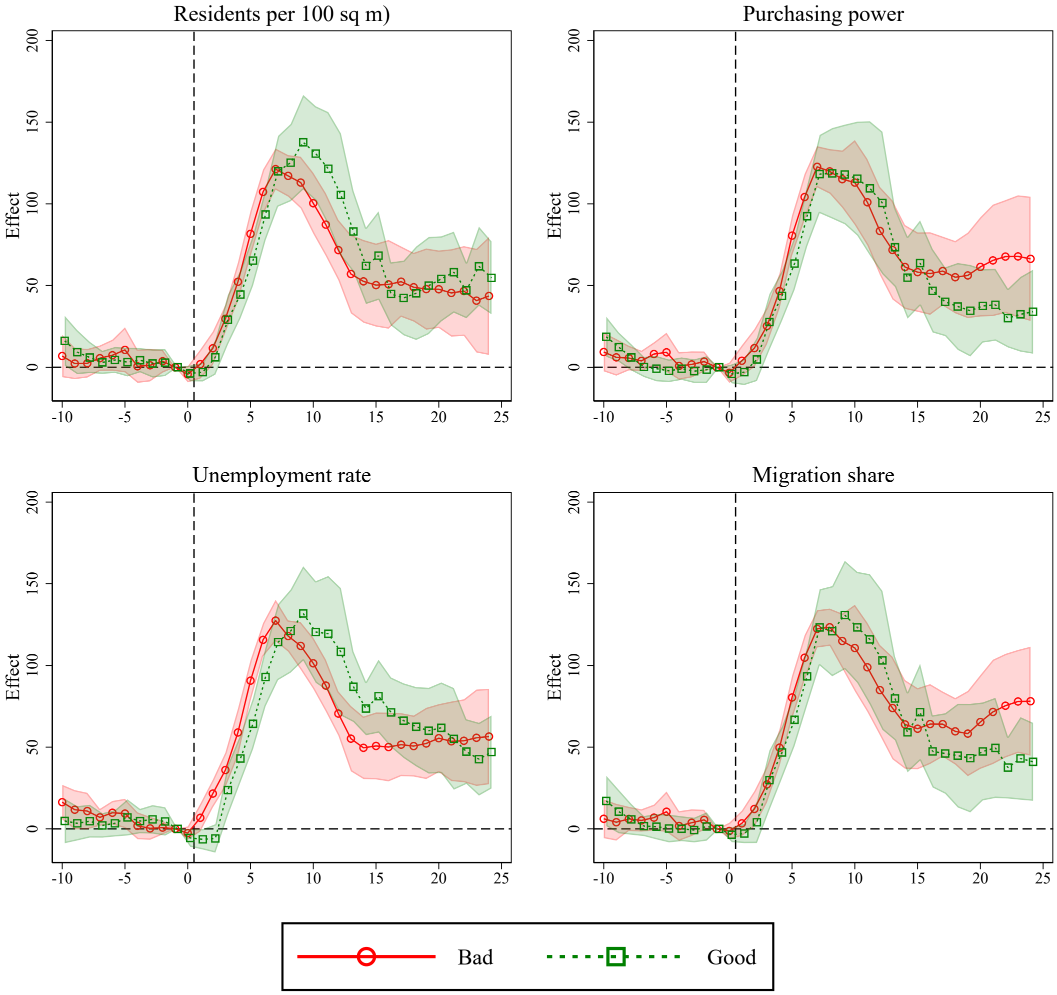

Event study results – Alternative measures of crowding and affluence. Coefficients corresponding to μ_j in Equation 3. μ_{-1} is restricted to zero. 95% confidence intervals reported. Standard errors clustered on district level. The figure was created using Stata 17 (https://www.stata.com/).

Figure S6

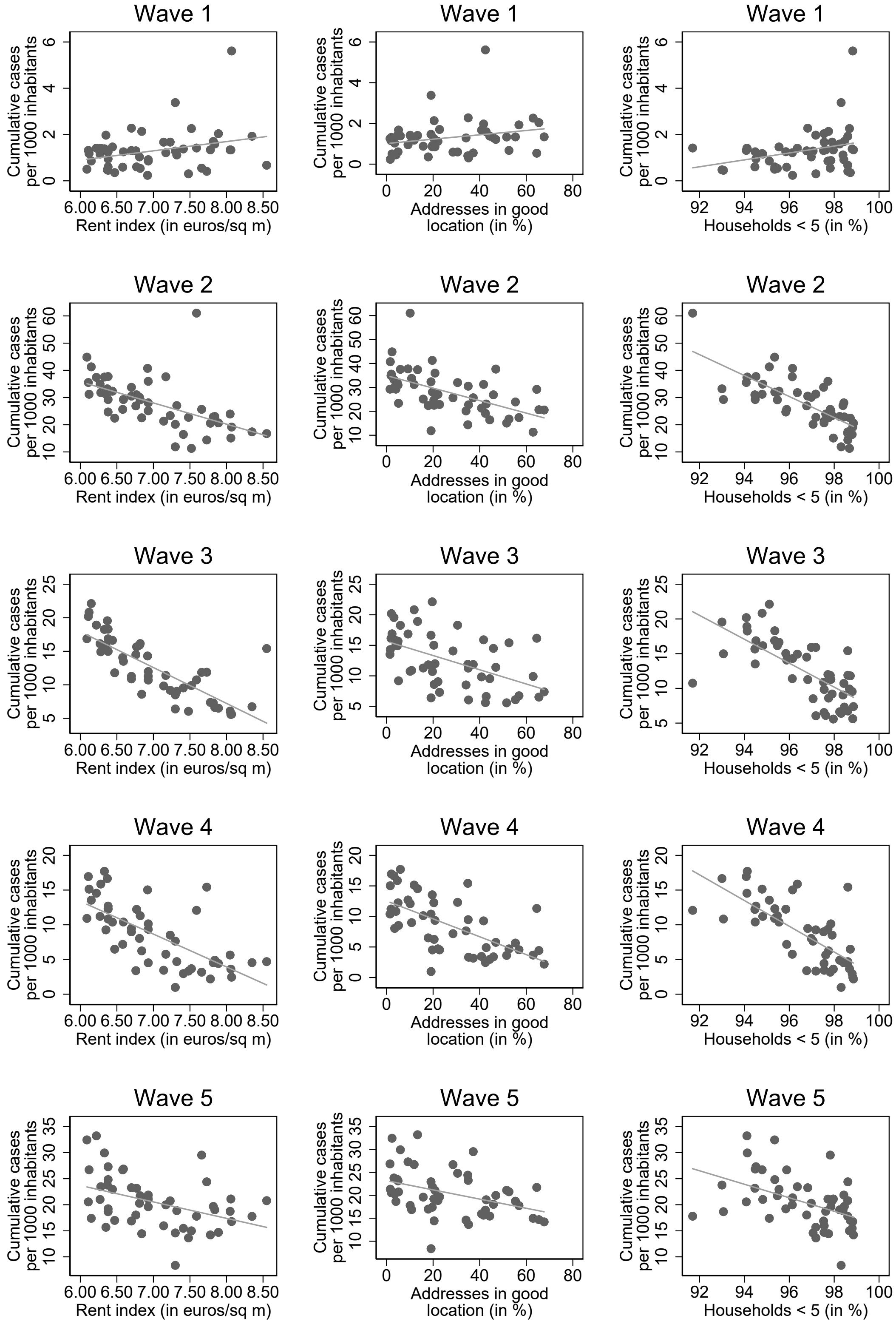

Cumulative cases and housing characteristics. The figure plots cumulative cases (per 1000 inhabitants) by wave against housing characteristics. Fitted values are based on bivariate linear regressions of cumulative cases on the respective housing measure. The figure was created using Stata 17 (`https://www.stata.com/`).
